# Screen Time Among Medical and Nursing Students and Its Correlation With Sleep Quality and Attention Span: A Cross-Sectional Study

**DOI:** 10.7759/cureus.58323

**Published:** 2024-04-15

**Authors:** Dinesh P Sahu, Manish Taywade, Poojitha Sushma Malla, Palak K Singh, Pratima Jasti, Pushpender Singh, Preeyal Sharma, Mukta Das, Ravi Bharathi U M, Krati Gupta

**Affiliations:** 1 Community Medicine and Family Medicine, All India Institute of Medical Sciences, Bhubaneswar, Bhubaneswar, IND

**Keywords:** digital health, pittsburg, students, medical, attention span, sleep, screen time

## Abstract

Background: Screen time is increasing among students and is also known to affect their lifestyle and health. The study investigated the correlation of screen time with sleep quality and attention span.

Methods: A cross-sectional study was conducted among undergraduate medical and nursing students in November 2021. A total of 192 students were selected randomly and investigated using a structured questionnaire. Sleep behavior was assessed using the Pittsburg Sleep Quality Index. Screen time and attention span were measured for each participant. The screen time data was reported as the median with an IQR. Pearson’s correlation was performed to assess the correlation between screen time and sleep behavior and screen time and attention span.

Results: The median screen time for 28 days was 260 (192.2-326.7) hours, and the median non-academic screen time was 250.0 (172.3-328.0) hours. Subjective sleep quality was good among 163 (84.9%) of the study participants. Global sleep quality was poor among 91 (42.2%) participants. The median score of the digit span forward was 6.00 (IQR: 5.00-7.00), and the median score of the digit span backward was 5.00 (IQR: 4.00-6.00). The global sleep score had a strong positive correlation with screen time, with a Pearson’s correlation coefficient of 0.86. Forward and backward attention spans were not correlated with sleep scores, with correlation coefficients of 0.037 and 0.071, respectively.

Conclusion: Screen time is increasing significantly among medical and nursing undergraduate students, and their sleep is also getting affected. Emphasis should be given to the balance between digitalization and health.

## Introduction

Human civilization has progressed and changed in the last few decades and has reprogrammed the very fabric of our lifestyles. Mobile phones and other electronic gadgets have become an integral part of our daily lives [1]. For a fraction of the population, their livelihood also revolves around these devices, increasing their exposure dramatically and giving rise to a wide range of medical conditions [2]. Screen time, as in the time spent using any form of these devices, was associated with an increased likelihood of metabolic syndrome in a dose-dependent manner, independent of physical activity [3]. High levels of screen time exposure are associated with an increased risk of migraine in young adults [4]. Screen time may also present as a risk factor or marker of anxiety and depression in adolescents [5]. Excessive digital media use by children and adolescents appears to be a significant factor that may hamper the formation of sound psychophysiological resilience [6].

Screen time and its effects on sleep were also studied earlier by many researchers. It has been seen that adolescents who used electronic media for six or more hours at night had higher odds of unhealthy eating behavior and inadequate sleep hours than those with two hours of use or less [7]. Hence, it can be deduced that screen time possibly has a significant effect on health-related parameters like sleep behavior, eating habits, obesity, etc.

Similarly, it was seen that sleep quality among medical students was poor [8]. Though numerous studies have been conducted on screen time and its related factors, there are still many gaps in our knowledge regarding this crucial new disruption in our lifestyles. Screen time affects various systems of our body. In our research, we have tried to find any possible correlation between screen time, attention span, and sleep among medical and nursing students. This population was chosen because the majority of students nowadays have one or more electronic gadgets. Many of them are also exposed to it for tens of hours a week. As soon-to-be medical professionals, they would be required to attend clinics and would have to pay attention to many life-saving and critical medical procedures and attend to patient complaints for extended periods every day, with their ability to provide adequate patient care non-waivered. Studies have proposed a possible relationship between cognition and sleep [9]. 

Screen time among youth is increasing, and medical and nursing students are not exempt from this change. The effect of screen time on sleep quality was assessed by researchers in other literature. However, literature was scarce on this aspect among Indian medical students. The relationship between screen time and attention span is not adequately studied. The study was conducted to find the correlation between screen time sleep behavior, and attention span among undergraduate medical and nursing students at the All India Institute of Medical Sciences, Bhubaneswar.

## Materials and methods

Study Site

The study was conducted at the All India Institute of Medical Sciences, Bhubaneswar, which is an institute of national importance (INI) located in the capital city of Odisha, an eastern state of India. The medical college provides medical, nursing, and paramedical courses. Being an INI, the students from the institute are from all the different parts of the country. Every year, 100 medical students and 100 nursing students are admitted to MBBS and BCS Nursing. The study was conducted between November 14 and November 21, 2021.

Study Participants

Undergraduate medical and nursing students aged 18 years or more were included in the study. Those who did not give consent and those who bought new mobile phones in the last 28 days were excluded from the study.

Sample Size

The sample size was calculated assuming a correlation coefficient of 0.2. The sample size calculated is 194. It was calculated using the following formula in nMaster software: Formula for sample size = N = [(Zα+Zβ)/C]2 + 3, where C = 0.5 * ln[(1+r)/(1-r)]. The sample was proportionally divided among all batches of medical and nursing undergraduate students. A line list was obtained from the institute authority. The participants were selected randomly from the sampling frame using random numbers generated in Microsoft Excel 2020.

Study Tool

A structured interview schedule was used for the study. The study tool included demographic characteristics, personal habits, a sleep questionnaire, and gadget details. The Pittsburgh Sleep Quality Index (PSQI) was used to assess sleep quality [10]. Attention span was measured by the digit span forward and digit span backward tests.

Study Procedure

The study was approved by the institute ethics committee of the All India Institute of Medical Sciences, Bhubaneswar (Ref Number: T/IM-NF/CM&FM/21/93). The participants were contacted after the academic hours. Written informed consent was obtained from the participants before the data collection. The participants were interviewed using the structured interview schedule. The screen time of the participants was assessed using electronic gadgets. For mobile phones, the screen time was recorded using the in-built screen time option in the phone settings. If a participant had more than one mobile phone or tablet, then all gadgets were considered to obtain screen time. For laptops and televisions, in the absence of clear data on screen time, recall from the participant was considered. The screen time for academic and non-academic purposes was obtained using the screen time for specific applications. Activities that fall under academic purposes include the use of e-learning, note-taking applications, and online eBooks. This data was available directly from the gadgets or also from the study application used for assessing screen time. Anything that did not fall under these categories was added under non-academic activities. All the screen times are recorded for the past 28 days from the day of the interview.

An attention span test was performed in a closed and silent room. Initially, the investigator showed a demo. First, the test was conducted with a three-digit span, where the participant was asked to repeat the sequence shown on the screen. The digit span increased gradually. The process was discontinued only when the participant could not repeat the number of digit sequences. Then, the digit span forward test was performed, followed by the digit span backward. In the digit span backward, the participant was asked to repeat the sequence in a reverse manner. A web-based version was used for the digit span to maintain uniformity in the data collected by different members involved in the study. The participant was asked to enter the data in a Google Form. All the other parameters, like socio-demographic, behavioral, and sleep quality, were recorded in the Google Form.

Statistical Analysis

Data collected in Google Forms was extracted into an Excel sheet. Data analysis was performed by IBM Corp. Released 2011. IBM SPSS Statistics for Windows, Version 20.0. Armonk, NY: IBM Corp. Categorical data was presented as proportions or percentages. Continuous data was presented as mean with standard deviation or median with interquartile range after testing for normality. Pearson correlation was used to assess the correlation between two continuous variables. A p-value of less than 0.05 was considered significant.

## Results

A total of 192 students participated in the study. Among them, 152 (79.2%) were medical undergraduate students, and 40 (20.2%) were nursing undergraduate students. Male and female participants were 96 (50%) each. Head of household: 87 (45.3%) participants were professionals by occupation, 94 (49.0%) were graduates, and 50 (26.0%) had professional or honors degrees. Only 12 (6.2%) students were tobacco users, and 25 (13%) students ever used alcohol products (Table 1). 

**Table 1:** Socio-demographic variables and substance use among the study participants (N=192)

Variables	Categories	Frequency (%)
Type of course	Medical	152 (79.2)
	Nursing	40 (20.2)
Gender	Male	96 (50.0)
	Female	96 (50.0)
Occupation of head of household	Professional	87 (45.3)
	Skilled workers and shop and market sales workers	25 (13.0)
	Legislators, Senior officials and managers	20 (10.4)
	Technicians and associate professionals	13 (6.8)
	Clerks	11 (5.7)
	Unemployed	11 (5.7)
	Elementary occupation	9 (4.9)
	Skilled agriculture and fishery workers	8 (4.2)
Education of head of the family	Graduate	94 (49.0)
	Profession or honours	50 (26.0)
	Intermediate	21(10.9)
	High school	18 (9.4)
	Middle school	9 (4.7)
Smoking	Never	180 (93.8)
	Ever	8 (4.2)
	Everyday	4 (2.1)
Alcohol consumption	Yes	25 (13.0)
	No	167 (87.0)

The median score of the digit span forward was 6.00 (IQR: 5.00-7.00), and the median score of the digit span backward was 5.00 (IQR: 4.00-6.00). The minimum and maximum scores for both forward and backward digit spans were 3 and 9, respectively (Table 2).

**Table 2:** Median screen times of the study participants (N=192) The data has been represented as medians with an interquartile range.

Screen time (in last 28 days)	Median (IQR)	
Mobile screen time (in Hours)	260.0 (192.2-326.7)	
Academic screen time for mobile (in Hours)	20.0 (4.0-53.0)	
Tablet/iPad screen time (in Hours)	40.0 (0.0-98.5)	
Academic screen time for Tablet/iPad (in Hours)	18.0 (0.0-58.0)	
Laptop screentime (in Hours)	0.0 (0.0-4.5)	
Academic screen time for Laptop (in Hours)	0.0 (0.0-1.0)	
Gaming device screen time (in Hours)	0.0 (0.0-0.0)	
Other devices screen time (in Hours)	0.0 (0.0-0.0)	
Total screen time (in Hours)	305.0 (222.3-385.0)	
Academic screen time in 28 days (in Hours)	35 (10.0-80.0)	
Non-academic screen time in 28 days (in Hours)	250.0 (172.3-328.0)	

All the study participants had at least one or more electronic gadgets. More than one electronic gadget was present in 120 (62.5%) of the study participants, and 29 (15.1%) study participants had more than two electronic gadgets. Among the study participants, 185 (96.4%) had the habit of using electronic devices like mobile phones before half an hour of sleep. The median total screen time, including all devices, in 28 days was 305 hours, with an IQR of 222.3-385.0 hours. Out of the total, mobile screen time was the major screen time, with a median screen time of 260 hours and an IQR of 192.2-326.7 hours, followed by iPads and tablets with a median duration of 40.0 hours and an IQR of 0.0-98.5 hours of usage. The median academic screen time in 28 days was 35 (10.0-80.0) hours. The majority of the screen time exposure was for non-academic causes, which was 250 (172.3-328.0) (Table 3). Total screen time, academic screen time, and non-academic screen time had no statistically significant association with gender, with p-values of 0.871, 0.193, and 0.677, respectively. 

**Table 3:** Types of screen time among the study participants (N=192) The data has been represented as frequency and percentages.

Variables	Categories	Frequency (%)
Using electronic gadgets	Yes	192 (100.0)
	No	0 (0.0)
Number of electronic gadgets	1	72 (37.5)
	2	91 (47.4)
	More than 2	29 (15.1)
Type of electronic gadget	Mobile	192 (100.0)
	Laptop	70 (36.4)
	Tablet/iPad	71 (36.9)
	Others*	4 (2.0)
Use devices within half an hour before sleep	Yes	185 (96.4)
	No	7 (3.6)

Sleep quality measured by PSQI was good among 111 (57.8%) study participants. Subjective sleep quality was very good among 63 (32.8%) of the study participants, and 100 (52.1%) considered it fairly good. Sleep latency was 15 minutes or less in 73 (38.0%) of the study participants. Sleeping more than 7 hours was reported in only 37 (19.3%). The habitual sleep efficiency was more than 85% in 128 (66.7%) of the study participants. However, habitual sleep efficiency was less than 65% in 13 (6.8%) of the study participants. Sleep disturbances in the last month were not reported by 20 (10.4%) of the study participants. Less than once a week, sleep disturbances were reported among 150 (78.1%) of the study participants. Only 2 (1%) participants reported having three or more sleep disturbances in a week. Sleep medication use was reported among 20 (12.5%) of the study participants. Among them, 4 (2.1%) used it three times or more in a week (Table 4).

**Table 4:** Sleep behavior among the study participants (N=192) The data has been presented as frequency and percentages.

Domain of sleep	Categories	Frequency (%)
Subjective sleep quality	Very good	63 (32.8)
	Fairly good	100 (52.1)
	Fairly bad	26 (13.5)
	Very bad	3 (1.6)
Sleep latency	≤ 15 minutes	73 (38.0)
	16-30 minutes	70 (36.5)
	31-60 minutes	35 (18.2)
	>60 minutes	14 (7.3)
Total sleep latency score	0	73 (38.0)
	1-2	70 (36.5)
	3-4	35 (18.2)
	5-6	14 (7.3)
Sleep duration	>7 hours	37 (19.3)
	6-7 hours	129 (67.2)
	5-6 hours	19 (9.9)
	<5hours	7 (3.6)
Habitual sleep efficiency	>85%	128 (66.7)
	75-84%	35 (18.2)
	65-74%	16 (8.3)
	<65%	13 (6.8)
Sleep disturbance	Not during past month	20 (10.4)
	Less than once a week	150 (78.1)
	Once or twice a week	20 (10.4)
	Three or more times a week	2 (1.0)
Sleep medication use	Not during past month	168 (87.5)
	Less than once a week	14 (7.3)
	Once or twice a week	6 (3.1)
	Three or more times a week	4 (2.1)
Day time sleep dysfunction	0	82 (42.7)
	1-2	79 (41.2)
	3-4	24 (12.6)
	5-6	7 (3.7)
Global PSQI	Good	111 (57.8)
	Poor	81 (42.2)

**Figure 1:**
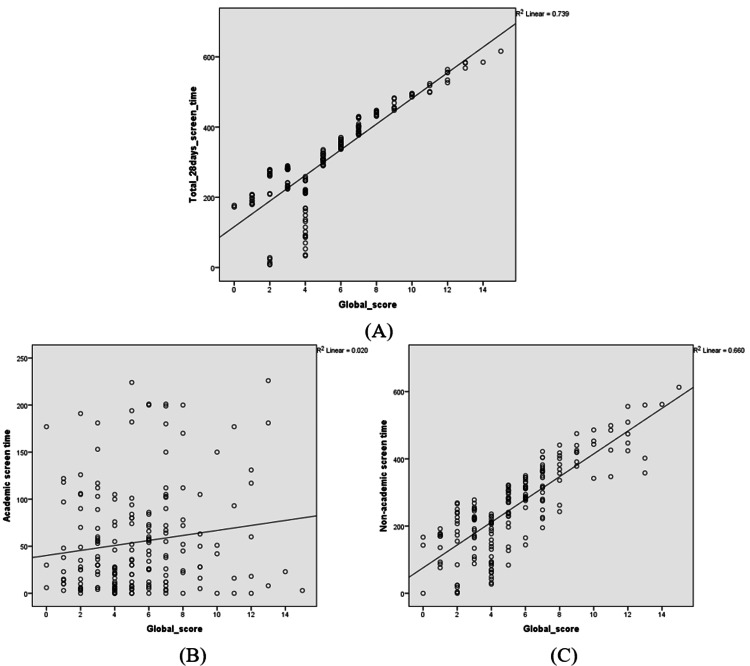
Correlation of screen time and sleep score (A: correlation between total screen time and global sleep score; B: correlation between academic screen time and global sleep score; C: correlation between non-academic screen time and global sleep score) The scatter plot has been represented by the correlation coefficient (r)

**Figure 2:**
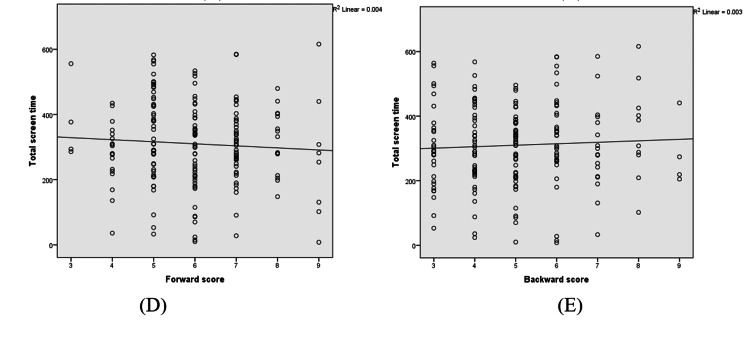
Correlation between screen time and attention span The scatter plot has been represented by the correlation coefficient (r). (D- Correlation between forward score and screen time, E- Correlation between backward score and screen time)

The global sleep score was strongly correlated with 28 days of total screen time, with a Pearson’s correlation coefficient of 0.86 and a p-value of <0.001. Similarly, non-academic screen time was also strongly correlated with global sleep score, with a correlation coefficient of 0.812 and a p-value of <0.001. The academic screen time was poorly correlated with the global sleep score. The correlation coefficient was 0.140, and the p-value was 0.050, which was insignificant. Forward and backward scores did not show any significant association with screen time. The forward score had a very weak negative or no correlation with a correlation coefficient of -0.066 (p=0.362), and the backward score had a weak positive correlation or no correlation with a correlation coefficient of 0.054 (p=0.461) (Figure 1). Similarly, no correlation was observed for the global score with the forward (coefficient=0.037, p=0.608) and backward scores (coefficient=-0.071, p=0.326) (Figure 2).

## Discussion

The study has reported the median screen time of the medical students in the study to be 305 hours in 28 days, with a screen time of 260 hours through mobile phones. Academic screen time (35 hours) was much less than non-academic screen time (250 hours). The mean digit span forward and backward was six (IQR: 5-7) and five (IQR: 4-6), respectively, with a relatively lesser backward digit span score. The sleep quality was poor among 42.2% of the study participants. The sleep score had a strong positive correlation with total screen time, re-enforcing the results found in different populations [11], and a similar correlation was observed for non-academic screen time.

A meta-analysis conducted by Rao et al. reported a 52.7% pooled prevalence of poor sleep quality [8]. The meta-analysis has included studies that used PSQI, similar to the current study. The reported prevalence of poor sleep quality in the meta-analysis was considerably higher. This could be due to the studies that included different populations, differences in sample size, and varied effect sizes. Another meta-analysis among Chinese medical students reported a 27.3% prevalence of poor sleep quality [12]. The prevalence of sleep disturbances in the Chinese meta-analysis could be due to the inclusion of one study population in the meta-analysis. A similar study from an Indian setting reported 66% of sleep disturbance among engineering students and 43% among medical students [13]. The result is quite similar to the current study.

Most of the studies conducted previously on screen time had a subjective assessment of screen time [14-16]. One of the studies by Christensen et al. obtained screen time using an objective method through a mobile application, similar to our study [17]. The median screen time reported in the study conducted in California was 38.4 hours. In the current study, we have found a median screen time of 305 hours. The result was noticeably higher in the current study as we have taken into account all types of electronic gadgets, whereas Christensen et al. only reported smartphone screen time. Sleep behavior was found to be associated with screen time, as reported in a few studies [18,19]. The association between sleep time, sleep onset latency, and sleep duration was reported in a study by Hjteland et al. among Norwegian university students [15]. Similarly, one Indian study also reported a significant association between different sleep behaviors and an increase in screen time [20]. Another study by Krishnan et al. reported an association between screen time and sleep latency, sleep duration, habitual sleep efficiency, and daytime sleep dysfunction [21]. In the current study, we have investigated screen time, including all of the electronic gadgets of the participants, in contrast to the available literature, where the investigators only included smartphones. In recent times, the use of iPads and tablets among medical and nursing undergraduate students has increased due to academic demand [22]. In the current study, 62.5% of the participants had more than one electronic gadget, and it was found that with an increase in the number of gadgets, screen time also significantly increased. Screen time is also reported to affect academic performance [23].

We measured attention span and analyzed for any significant association with screen time. There had been no previous literature or work done to discover an association between screen time and attention span. Although some studies have shown a more specific association between internet usage and attention in some populations [24], Another study conducted by Stefansdottir et al. did not find any significant association between sleep behavior and memory and attention in healthy adolescents [25]. The results were similar to our study, where we did not find any association between attention span, sleep behavior, or screen time. The results also concluded that our hypothesis of an affected attention span due to sleep behavior and screen time was not true. The visual attention span is important as it is a measure of the working memory of the individual, affecting their daily activities, and in our given population, it could have an additional implication in the context of patient care. In the current study, despite sleep disturbances, the attention span was not affected.

The study was the first of its kind in eastern India, undertaken to assess the association between attention span and screen time. The study was conducted among medical and nursing undergraduate students of an institute of national importance, where the students were from different parts of the country. The greatest strength of the study is the objective assessment of screen time, which was used only in a few previous studies. However, the study has several limitations. Sleep quality is known to likely be affected by substance use. This substance use information was included in our study, but it was self-reported, and there is always a chance of social desirability bias for substance use. There were several confounders, like physical activity, food consumption patterns, and body mass index, that were not included in the study. The sample size could not be reached; however, back calculation has shown that the power is sufficient enough to provide a conclusive result. Additionally, since the study is a cross-sectional study, the causal relationship cannot be established.

## Conclusions

The screen time of medical and nursing undergraduate students is increasing significantly in current times, and its impact on sleep behavior is noticeable. Screen time is adversely affecting subjective sleep quality, sleep latency, and sleep efficiency. Intervention is required to reduce screen time, increase physical activity among adolescents and young adults, and change patterns of teaching and training. This study could not confirm the association between screen time and attention span. With several sectors of our world becoming digitized, the education system is changing too. We need to stress a healthy balance between digitalization and health.

## References

[1] Marques O (2016). Innovative technologies in everyday life. Spr Inter Publ.

[2] Hegde AM, Suman P, Unais M, Jeyakumar C (2019). Effect of electronic gadgets on the behaviour, academic performance and overall health of school going children-a descriptive study. Jr Adv Med Den Sci Res.

[3] Mark AE, Janssen I (2008). Relationship between screen time and metabolic syndrome in adolescents. J Public Health (Oxf).

[4] Montagni I, Guichard E, Carpenet C, Tzourio C, Kurth T (2016). Screen time exposure and reporting of headaches in young adults: A cross-sectional study. Cephalalgia.

[5] Maras D, Flament MF, Murray M, Buchholz A, Henderson KA, Obeid N, Goldfield GS (2015). Screen time is associated with depression and anxiety in Canadian youth. Prev Med.

[6] Lissak G (2018). Adverse physiological and psychological effects of screen time on children and adolescents: Literature review and case study. Environ Res.

[7] Cha EM, Hoelscher DM, Ranjit N (2018). Effect of media use on adolescent body weight. Prev Chronic Dis.

[8] Rao WW, Li W, Qi H (2020). Sleep quality in medical students: a comprehensive meta-analysis of observational studies. Sleep Breath.

[9] Wild CJ, Nichols ES, Battista ME, Stojanoski B, Owen AM (2018). Dissociable effects of self-reported daily sleep duration on high-level cognitive abilities. Sleep.

[10] Smyth C (1999). The Pittsburgh sleep quality index (PSQI). J Gerontol Nurs.

[11] Wu X, Tao S, Zhang Y, Zhang S, Tao F (2015). Low physical activity and high screen time can increase the risks of mental health problems and poor sleep quality among Chinese college students. PLoS One.

[12] Sun Y, Wang H, Jin T, Qiu F, Wang X (2022). Prevalence of sleep problems among Chinese medical students: a systematic review and meta-analysis. Front Psychiatry.

[13] Baby RS, Issac A, Vasudev A (2021). Impact of screen time on sleep quality. Indian J Psy Nsg.

[14] Foerster M, Henneke A, Chetty-Mhlanga S, Röösli M (2019). Impact of adolescents’ screen time and nocturnal mobile phone-related awakenings on sleep and general health symptoms: A prospective cohort study. Int J Environ Res Public Health.

[15] Hjetland GJ, Skogen JC, Hysing M, Sivertsen B (2021). The association between self-reported screen time, social media addiction, and sleep among Norwegian university students. Front Public Health.

[16] Mao Y, Xie B, Chen B (2022). Mediating effect of sleep quality on the relationship between electronic screen media use and academic performance among college students. Nat Sci Sleep.

[17] Christensen MA, Bettencourt L, Kaye L (2016). Direct measurements of smartphone screen-time: Relationships with demographics and sleep. PLoS One.

[18] Al-Anazi NS, Al-Harbi Z (2022). Association of electronic media use and sleep habits among secondary school students in Al-Madinah. Cureus.

[19] Naito R, Yun Low W, Wan Yuen C (2021). Sleep deprivation and its associated factors among undergraduate students in Malaysia. Asia Pac J Public Health.

[20] Gupta PC, Rana M, Ratti M (2022). Association of screen time, quality of sleep and dry eye in college-going women of Northern India. Indian J Ophthalmol.

[21] Krishnan B, Sanjeev RK, Latti RG (2020). Quality of sleep among bedtime smartphone users. Int J Prev Med.

[22] Singh K, Sarkar S, Gaur U, Gupta S, Adams OP, Sa B, Majumder MA (2021 Nov 2). Smartphones and educational apps use among medical students of a smart university campus. Frontiers in Communication.

[23] Rathakrishnan B, Bikar Singh SS, Kamaluddin MR, Yahaya A, Mohd Nasir MA, Ibrahim F, Ab Rahman Z (2021). Smartphone addiction and sleep quality on academic performance of university students: An exploratory research. Int J Environ Res Public Health.

[24] Kuo SY, Chen YT, Chang YK, Lee PH, Liu MJ, Chen SR (2018). Influence of internet addiction on executive function and learning attention in Taiwanese school-aged children. Perspect Psychiatr Care.

[25] Stefansdottir R, Gundersen H, Rognvaldsdottir V (2020). Association between free-living sleep and memory and attention in healthy adolescents. Sci Rep.

